# Association of meeting 24-hour movement guidelines with low back pain among adults

**DOI:** 10.3934/publichealth.2023062

**Published:** 2023-11-24

**Authors:** Kaja Kastelic, Nejc Šarabon, Michael D. Burnard, Dean Lipovac, Željko Pedišić

**Affiliations:** 1 Andrej Marušič Institute, University of Primorska, Muzejski trg 2, 6000 Koper, Slovenia; 2 InnoRenew CoE, Livade 6a, 6310 Izola, Slovenia; 3 Faculty of Health Sciences, University of Primorska, Polje 42, 6310 Izola, Slovenia; 4 Institute for Health and Sport, Victoria University, Building P, Footscray Park Campus, Ballarat Road, Footscray VIC 3011, Melbourne, Australia

**Keywords:** musculoskeletal health, recommendations, movement behaviors, physical behaviors, time-use epidemiology, Daily Activity Behaviours Questionnaire

## Abstract

**Background:**

According to recently published 24-hour movement guidelines, adults should spend: ≥150 minutes/week in moderate-to-vigorous physical activity (MVPA); <8 hours/day in sedentary behaviour (SB); and 7–9 hours/day sleeping.

**Objective:**

We explored the association between meeting these recommendations and low back pain (LBP)—the most common musculoskeletal disorder.

**Methods:**

We collected self-reported data from 2333 adults about: MVPA, SB and sleep duration; frequency and intensity of LBP; and sociodemographic and lifestyle characteristics.

**Results:**

Meeting a combination of SB and sleep recommendations was associated with lower odds of LBP in the past week and past month (adjusted odds ratio [*OR*]: 0.64 and 0.52, respectively; *p* < 0.05 for both). Among LBP sufferers, meeting any combination of recommendations that includes sleep was associated with lower odds of frequent (*OR* range: 0.49–0.61; *p* < 0.05 for all) and intense (*OR* range: 0.39–0.66; *p* < 0.05 for all) LBP in the past week, while meeting a combination of SB and sleep recommendations or all three recommendations was associated with lower odds of intense LBP in the past month and past year (*OR* range: 0.50–0.68; *p* < 0.05 for all). The likelihood of experiencing higher frequency and intensity of LBP decreased with the number of recommendations met (*p* for linear trend < 0.05).

**Conclusion:**

Meeting the SB and sleep recommendations in combination is associated with a lower likelihood of LBP, while adhering to the overall 24-hour movement guidelines or any combination of recommendations that includes sleep is associated with lower frequency and intensity of LBP among LBP sufferers.

## 1. Introduction

Low back pain (LBP) is the most common musculoskeletal disorder. It affects around 38% of the global adult population each year [1], and it is among the leading causes of disability [2],[3]. LBP has an adverse effect on the quality of life [4],[5], and it presents a substantial economic burden for society. The estimated annual direct medical costs and indirect costs (e.g., due to absenteeism, presentism and early retirement) of back pain may be as high as $868.4 per capita [6], accounting for around 0.8%–2.1% of the gross domestic product in high-income countries [7]. In terms of the economic impact, LBP is comparable to cardiovascular disease [8],[9], diabetes [10] and cancer [11].

Numerous potential risk factors for the development, persistence and aggravation of LBP have been studied so far, including physical, psychological and social factors [12]–[15]. The aetiology of LBP is multi-factorial and complex [16]. A single risk factor usually has very limited explanatory value, and a combination of different factors was proposed to have a synergistic effect [16]. However, a review on the prevention of LBP development among adults reported that exercise is the only evidence-based and effective prevention strategy [17]. Also, the clinical practice guidelines for the management of LBP recommend that first-line treatment should include exercise therapy, education, cognitive behavioural therapy, and advice to stay active, avoid bed rest and keep healthy sleep habits [18]–[22].

Current recommendations for the prevention and management of LBP emphasise the importance of movement and non-movement behaviours. It is recommended for LBP sufferers to engage in structured and incidental physical activity, avoid sedentary behaviour (SB) and maintain healthy sleep habits. These three movement behaviours—physical activity, SB and sleep—are time-use components of the 24-hour day [23],[24]. They are mutually exclusive (you can only engage in one of these behaviours at a time), exhaustive (their sum will always be equal to 24 hours per day) and consequently perfectly collinear components (i.e., change in one behaviour will lead to a proportional change in other behaviours), indicating they should be considered in combination. However, the current guidelines for the prevention and management of LBP do not provide recommendations on how much time should be optimally spent in each of the movement behaviours.

Recently, public health guidelines for adults that integrate recommendations on time spent in physical activity, SB and sleep have been issued [25]–[28]. The guidelines acknowledge that movement behaviours collectively impact health and well-being. They are referred to as “24-hour movement guidelines”, and they provide quantitative recommendations on the time spent in moderate-to-vigorous physical activity (MVPA), SB and sleep. For example, the Canadian 24-hour movement guidelines for adults recommend at least 150 minutes of MVPA per week, no more than eight hours of SB per day and seven to nine hours of sleep per day [25].

It is unclear whether meeting the 24-hour movement guidelines is associated with the frequency and intensity of LBP. Therefore, the aim of this study was to explore the associations of meeting different combinations of 24-hour movement guidelines with the frequency and intensity of LBP among adults. We hypothesized that meeting the 24-hour movement guidelines is favourably associated with the occurrence, frequency and intensity of LBP.

## 2. Methods

### 2.1. Study design and participants

Cross-sectional data on (1) time spent in MVPA, SB and sleep; (2) frequency and intensity of LBP within the previous year; and (3) sociodemographic and lifestyle variables were collected using an online survey. Participants were recruited among Slovenian residents aged 18 years and over via mailing lists, daily newspapers, web-portals and social media. After removing 110 participants with missing data, the final sample in this study included 2333 participants (74% females) with the mean (± standard deviation) age of 48 ± 14 years (Table 1). All participants provided informed consent before responding to the survey. The study protocol was approved by the National Medical Ethics Committee (ref: 0120–557/2017/4). More details about the study design and participants were reported elsewhere [29].

### 2.2. Measures

#### 2.2.1. Movement behaviours

Data on 24-hour movement behaviours were assessed using the Daily Activity Behaviours Questionnaire (DABQ). This questionnaire asks about time spent in MVPA, SB and sleep in the past seven days. The questionnaire has shown acceptable test-retest reliability (ICC = 0.59–0.65) and validity against activPAL accelerometer (*ρ* = 0.38–0.66) for the MVPA, SB and sleep estimates [30],[31]. Participants were categorised according to the combination of recommendations from the Canadian 24-hour movement guidelines for adults and older adults they adhere to. The guidelines include the following recommendations: (1) engage in MVPA for at least 150 minutes per week; (2) spend no more than eight hours per day in SB; and (3) sleep seven to nine hours per day (for adults) or seven to eight hours per day (for older adults) [25]. The participants were categorised into one of the following groups: (1) not meeting any of the recommendations; (2) meeting only the recommendation for MVPA; (3) meeting only the recommendation for SB; (4) meeting only the recommendation for sleep; (5) meeting the MVPA and SB recommendations; (6) meeting the MVPA and sleep recommendations; (7) meeting the SB and sleep recommendations; and (8) meeting the MVPA, SB and sleep recommendations.

#### 2.2.2. Low back pain

The outcome measures were experiencing/not experiencing LBP, frequency of LBP (i.e., on how many days) and intensity of LBP (i.e., how strong). The question: “*On how many days have you experienced low back pain in the last 12 months?*” with the following response options: *0 days / 1–7 days / 8–30 days / 31–90 days / more than 90 days, but not every day / every day* [32] was used to assess whether the participants experienced (i.e., any response other than *0 days*) or did not experience (*0 days*) LBP in the past year. The participants who experienced LBP in the past year were further categorised into the following groups: *1–30 days, 31–90 days and more than 90 days* with LBP during the past year [33],[34].

The participants who experienced LBP in the past year were additionally asked about their LBP intensity in a given period using the following questions: “*How would you rate the average intensity of your low back pain during the last 12 months (average pain intensity on days when you experienced pain)?*”, “*How intense was the worst low back pain that you experienced in the last month?*” and “*How intense was the worst low back pain that you experienced in the last week?*” The responses were provided on a visual analogue scale (VAS) ranging from no pain to the worst pain imaginable (range of scores from 0 to 100) [35],[36]. The LBP intensity was categorised using the following cut-off points: 0 for no pain, 1–38 for mild pain, 39–57 for moderate pain and 58–100 for severe pain [37]. The questions on LBP intensity in the past month and past week were used to assess whether the participants experienced (i.e., any response other than VAS = 0) or did not experience (VAS = 0) LBP in the past month and past week.

#### 2.2.3. Sociodemographic and lifestyle variables

The following sociodemographic and lifestyle variables were assessed and adjusted for in the analysis: age (categorised as *18 to 44 years / 45 to 64 years / 65 years or more*, based on the classification defined by the Medical Subject Headings, to allow for possible non-linear relationships with the outcome variables), sex (*female / male*), smoking status (*smoking occasionally or every day / quit smoking or never smoked*), experiencing stress (*often or every day / occasionally, very rarely, or never*), level of education (*primary or secondary education / higher education*) and self-reported overall socio-economic status (*high or very high / middle / low or very low*). Body mass index (BMI) was calculated from the self-reported body height and weight and categorised as *underweight or “normal” weight* [< 25 kg/m^2^] and *overweight or obese* [≥25.0 kg/m^2^]. These covariates were chosen based on the published literature on their association with movement behaviours [29],[38],[39] and LBP [40]–[42].

### 2.3. Statistical analysis

The data were analysed using R version 4.0.5 [43] and R Studio 1.4.1106 [44] using the packages *brant* [45], *DHARMa* [46], *dplyr* [47], *generalhoslem* [48], *janitor* [49], *MASS* [50], *rstatix* [51] and *skimr* [52]. Participant characteristics were presented as absolute and relative frequencies. The associations of meeting the 24-hour movement guidelines with experiencing/not experiencing LBP in the past year, month and week were analysed using a series of binary logistic regressions. Furthermore, the associations of meeting the 24-hour movement guidelines with the frequency and intensity of LBP were analysed using a series of ordinal logistic regression (proportional odds) analyses. These analyses were restricted to LBP sufferers.

All analyses were adjusted for age, sex, BMI, smoking, stress, education and socio-economic status. The assumptions and goodness of fit of the models were tested as follows: (1) for the binary logistic model, residuals were analysed using a simulation-based approach with tests for distribution, dispersion and outliers [46]; and (2) for the ordinal logistic models, the Brant [53], Hosmer-Lemeshow test, Lipsitz and Pulkstenis-Robinson tests [54] were performed. The tests confirmed that the assumptions were not violated, and that goodness of fit was acceptable for all models.

## 3. Results

### 3.1. Sample characteristics

Most participants had middle socio-economic status and were non-smokers, highly educated and employed. Approximately half of the participants were overweight or obese. The prevalence of not meeting any of the 24-hour movement recommendations was 7%. A single 24-hour movement recommendation was met by 26% of participants, two were met by 40% of participants and all three were met by 25% of participants. The prevalence of LBP in the past year, past month and past week was 71%, 59% and 46%, respectively. Among the LBP sufferers (i.e., those who experienced LBP in the past year), most participants experienced LBP on 1 to 30 days in the past year (Supplementary Table S1).

### 3.2. Meeting 24-hour movement guidelines and experiencing low back pain

In the binary logistic regression, we did not find a significant association between meeting the 24-hour movement guidelines and experiencing LBP in the past year (Table 2). Those who met a combination of SB and sleep recommendations were less likely to experience LBP in the past month (*OR* = 0.64, 95% *CI*: 0.43, 0.95) and past week (*OR* = 0.52, 95% *CI*: 0.35, 0.76), compared with those who did not meet any of the 24-hour movement recommendations. Detailed results of these analyses are provided in Supplementary Tables S2, S3 and S4.

The binary logistic regression analysis with the number of guidelines met as an explanatory variable revealed that those who met a combination of two recommendations were less likely to experience LBP in the past week (*OR* = 0.68, 95% *CI*: 0.49, 0.95). We did not find a significant linear trend for any of the LBP variables presented in Table 2.

### 3.3. Meeting 24-hour movement guidelines and frequency and intensity of low back pain

Among the LBP sufferers (i.e., those who experienced LBP in the past year), the ordinal logistic regression analysis revealed that those who met a combination of MVPA and sleep, SB and sleep or all three recommendations were less likely to report a higher frequency of LBP in the past year, compared with those who did not meet any of the recommendations (Table 3). Those who met a combination of SB and sleep or all three recommendations were less likely to report a higher intensity of LBP in the past year and past month. Those who met a combination of MVPA and sleep, SB and sleep, or all three recommendations were less likely to report a higher intensity of LBP in the past week. Detailed results of these analyses are provided in Supplementary Tables S5, S6, S7 and S8.

**Table 1.** Participant characteristics (total sample, *n* = 2333; non-LBP sufferers, *n* = 673; LBP sufferers, *n* = 1660).

**Characteristic**	**Total sample** ***n* (%)**	**Non-LBP sufferers** ***n* (%)**	**LBP sufferers** ***n* (%)**
**Age group**			
18 to 44 years	896 (38)	298 (44)	598 (36)
45 to 64 years	1153 (49)	293 (44)	860 (52)
65 years or more	284 (12)	82 (12)	202 (12)
**Sex**			
Female	1731 (74)	506 (75)	1225 (74)
Male	602 (26)	167 (25)	435 (26)
**BMI**			
Underweight or “normal” weight (<25 kg/m^2^)	1220 (52)	393 (58)	827 (50)
Overweight or obese (≥25.0 kg/m^2^)	1113 (48)	280 (42)	833 (50)
**Smoking status**			
Smoking occasionally or every day	404 (17)	106 (16)	296 (18)
Quit smoking or never smoked	1929 (83)	567 (84)	1364 (82)
**Experiencing stress**			
Often or every day	828 (35)	201 (30)	627 (38)
Occasionally, very rarely or never	1505 (65)	472 (70)	1033 (62)
**Education**			
Primary or secondary education	687 (30)	158 (23)	529 (32)
Higher education	1646 (70)	515 (77)	1131 (68)
**Socio-economic status**			
High or very high	277 (12)	105 (16)	172 (10)
Middle	1820 (78)	521 (77)	1299 (78)
Low or very low	236 (10)	47 (7)	189 (11)
**Meeting guidelines**			
None	171 (7)	42 (6)	129 (8)
Only for MVPA	221 (9)	61 (9)	160 (10)
Only for SB	175 (8)	52 (8)	123 (7)
Only for sleep	217 (9)	68 (10)	149 (9)
For MVPA and SB	279 (12)	82 (12)	197 (12)
For MVPA and sleep	370 (16)	123 (18)	247 (15)
For SB and sleep	309 (13)	86 (13)	223 (13)
For MVPA, SB and sleep	591 (25)	159 (24)	432 (26)

Note: LBP = low back pain; BMI = body mass index; MVPA = moderate-to-vigorous physical activity; SB = sedentary behaviour.

The ordinal logistic regression analysis with the number of guidelines met as an explanatory variable revealed that the LBP sufferers who met a combination of two or all three 24-hour movement recommendations were less likely to report a higher frequency of LBP in the past year, higher average intensity of LBP in the past year and higher intensity of LBP in the past week. Those who met all three 24-hour movement recommendations were less likely to report a higher intensity of LBP in the past month. The linear trend was significant for all LBP variables presented in Table 3, indicating that the likelihood of higher frequency and intensity of LBP decreases with the number of guidelines met.

**Table 2.** Associations between meeting different combinations of 24-hour movement guidelines and experiencing low back pain in the past year, month and week (*n* = 2333).

**Project**	**LBP in the past year** ***OR* [95% *CI*]**	**LBP in the past month** ***OR* [95% *CI*]**	**LBP in the past week** ***OR* [95% *CI*]**
**Guideline(s) met**			
None	[*ref*]	[*ref*]	[*ref*]
Only for MVPA	0.90 [0.56, 1.43]	0.83 [0.54, 1.27]	0.85 [0.56, 1.28]
Only for SB	0.77 [0.47, 1.25]	0.71 [0.45, 1.10]	0.71 [0.46, 1.09]
Only for sleep	0.82 [0.52, 1.30]	0.80 [0.52, 1.22]	0.83 [0.55, 1.26]
For MVPA and SB	0.86 [0.55, 1.34]	0.82 [0.54, 1.23]	0.84 [0.57, 1.25]
For MVPA and sleep	0.80 [0.52, 1.21]	0.84 [0.57, 1.24]	0.73 [0.50, 1.06]
For SB and sleep	0.93 [0.60, 1.43]	0.64 [0.43, 0.95]*	0.52 [0.35, 0.76]**
For MVPA, SB and sleep	1.04 [0.69, 1.54]	0.89 [0.61, 1.28]	0.83 [0.58, 1.19]
**Number of guidelines met**			
0	[*ref*]	[*ref*]	[*ref*]
1	0.83 [0.56, 1.23]	0.78 [0.54, 1.12]	0.80 [0.56, 1.13]
2	0.86 [0.58, 1.25]	0.76 [0.54, 1.08]	0.68 [0.49, 0.95]*
3	1.04 [0.69, 1.54]	0.89 [0.61, 1.28]	0.83 [0.58, 1.18]
*p*-value for linear trend	0.833	0.513	0.194

Note: LBP = low back pain; OR = adjusted odds ratio from a binary logistic regression analysis; CI = confidence interval; MVPA = moderate-to-vigorous physical activity; SB = sedentary behaviour. All models were adjusted for age, sex, body mass index, smoking, stress, education and socio-economic status. *p  <  0.05, **p  <  0.01.

## 4. Discussion

### 4.1. Main findings

The main finding of our study is that meeting the 24-hour movement guidelines is favourably associated with LBP among the LBP sufferers. Specifically, meeting a combination of MVPA and sleep recommendations, a combination of SB and sleep recommendations or all three recommendations is associated with lower odds of experiencing higher frequency and intensity of LBP. The likelihood of experiencing higher frequency and intensity of LBP decreases with the number of recommendations met. Furthermore, while meeting the combination of SB and sleep recommendations was associated with lower odds of experiencing LBP, no such associations were found for meeting the MVPA recommendation or any combination of recommendations that included MVPA.

### 4.2. Comparison with previous studies

Studies on the relationships between movement behaviours and LBP mostly reported that moderate and high levels of MVPA are associated with a lower risk of experiencing LBP [55],[56], and that lower sleep duration is associated with higher odds of LBP [57],[58]. Studies on the association between SB and LBP produced inconsistent results, showing either positive association [59],[60] or no association [61],[62]. If we would hypothesise a causal relationship between MVPA as predictor and LBP as outcome variable, it might be that the dose of MVPA among the participants in our study who met the MVPA recommendation was not high enough to reduce the likelihood of experiencing LBP. This would suggest that simply meeting the MVPA recommendation may not necessarily be sufficient to help prevent LBP. To test this assumption, future studies should consider different doses of MVPA that are higher than the threshold for meeting the MVPA recommendation. The observed disagreement with previous findings might also be due to the differences in the definition of LPB. In our study, LBP was defined as experiencing any LBP in the past year, month or week, while some previous studies specified the minimal severity threshold. Another reason may be the difference in the analytical approach. Our analyses included all three movement behaviours, while most other studies explored the associations of LBP with a single movement behaviour while inadequately accounting for the remaining movement behaviours.

**Table 3.** Associations of meeting different combinations of 24-hour movement guidelines with the frequency and intensity of low back pain (*n* = 1660).

**Project**	**LBP frequency in the past year**	**Average LBP intensity in the past year**	**Highest LBP intensity in the past month**	**Highest LBP intensity in the past week**
	***OR* [95% *CI*]**	***OR* [95% *CI*]**	***OR* [95% *CI*]**	***OR* [95% *CI*]**
**Guideline(s) met**				
None	[*ref*]	[*ref*]	[*ref*]	[*ref*]
Only for MVPA	0.80 [0.49, 1.31]	0.90 [0.57, 1.42]	0.86 [0.56, 1.32]	0.81 [0.53, 1.26]
Only for SB	0.84 [0.50, 1.42]	0.80 [0.49, 1.29]	0.87 [0.55, 1.37]	0.77 [0.49, 1.24]
Only for sleep	0.79 [0.48, 1.32]	0.66 [0.41, 1.06]	0.86 [0.55, 1.33]	0.84 [0.54, 1.30]
For MVPA and SB	0.81 [0.50, 1.31]	0.77 [0.50, 1.20]	0.93 [0.61, 1.41]	0.79 [0.52, 1.19]
For MVPA and sleep	0.49 [0.30, 0.79]**	0.73 [0.48, 1.11]	0.88 [0.60, 1.31]	0.66 [0.44, 0.98]*
For SB and sleep	0.61 [0.38, 0.99]*	0.52 [0.34, 0.81]**	0.50 [0.33, 0.76]**	0.39 [0.26, 0.59]***
For MVPA, SB and sleep	0.61 [0.40, 0.95]*	0.64 [0.44, 0.95]*	0.68 [0.47, 0.99]*	0.61 [0.42, 0.89]*
**Number of guidelines met**				
0	[*ref*]	[*ref*]	[*ref*]	[*ref*]
1	0.81 [0.53, 1.24]	0.79 [0.54, 1.16]	0.86 [0.60, 1.25]	0.81 [0.56, 1.18]
2	0.62 [0.41, 0.94]*	0.67 [0.47, 0.97]*	0.75 [0.53, 1.06]	0.59 [0.41, 0.84]**
3	0.61 [0.39, 0.94]*	0.65 [0.44, 0.96]*	0.69 [0.48, 1.00]*	0.62 [0.43, 0.90]*
*p*-value for linear trend	0.010	0.017	0.028	0.002

Note: LBP = low back pain; OR = adjusted odds ratio from an ordinal logistic regression analysis; CI = confidence interval; MVPA = moderate-vigorous physical activity; SB = sedentary behaviour; All models were adjusted for age, sex, body mass index, smoking, stress, education and socio-economic status. The analyses included only LBP sufferers. *p  <  0.05, **p  <  0.01, ***p  <  0.001

Previous studies suggested that more MVPA [56],[63], less SB [61],[64] and higher sleep duration [65],[66] are associated with lower frequency and intensity of LBP among the LBP sufferers. Our findings did not confirm that any of these behaviours is individually associated with LBP. Instead, our findings suggest that meeting any combination of recommendations that includes sleep is associated with lower frequency and intensity of LBP. Most studies to date explored the associations of LBP with a single movement behaviour while inadequately accounting for the remaining movement behaviours, which may explain why our results did not corroborate their findings. Our findings suggest that a single “unhealthy” movement behaviour may not necessarily be an issue in regard to LBP. For example, it is possible that being sedentary for more than eight hours per day is not unfavourably associated with LBP, if it is compensated by adequate amounts of MVPA and sleep.

We did not find a significant association of meeting the MVPA recommendation alone with the frequency and intensity of LBP. This might be misinterpreted as inconsistent with the current body of evidence showing the importance of physical activity for LBP [56],[63]. However, the frequency and intensity are not the only important LBP outcomes that need to be examined in relation to physical activity. For example, a recent prospective study conducted among Danish workers found that increasing leisure-time MVPA by 20 minutes per day is associated with a 26% lower risk of long-term sickness absence among the LBP sufferers [67]. It is possible that MVPA does not help reduce the frequency and intensity of LBP, but only enables the LBP sufferers to better cope with the pain. Furthermore, the MVPA recommendation does not differentiate between different domains (work, household, transport and leisure time) and types (e.g., exercise, gardening) of physical activity. It may be that only some domains of physical activity are favourably associated with LBP. For example, in the above-mentioned Danish study, in contrast to MVPA in leisure-time, increasing occupational MVPA by 20 minutes was associated with a 38% higher risk of long-term sickness absence [67]. Studies also show that different types of MVPA have different associations with LBP; while some types of MVPA were shown to be beneficial (e.g., brisk walking, exercise) [63],[68]–[71], others might be detrimental (e.g., physical activities that include frequent and repetitive lifting of high loads) [62],[72],[73]. Given that all domains and types of MVPA contribute towards meeting the MVPA recommendation, it may be that in our analysis their differing associations with LBP balanced each other out.

### 4.3. Practical implications

If we would hypothesise a causal relationship between movement behaviours (as predictors) and LBP (as outcome), our findings would suggest that the adherence to the 24-hour movement guidelines should be considered as a component of self-management and clinical strategies for management of LBP. Our findings would also suggest that LBP sufferers would benefit from sleeping between seven and nine hours per day and limiting SB to no more than eight hours per day. Moreover, meeting the 24-hour movement guidelines would likely help them manage their comorbid health conditions, which would improve their overall health and further strengthen their ability to manage LBP [74],[75] and limit the impact of LBP on their quality of life [76]. Observed reductions in the odds of higher frequency and intensity of LBP associated with meeting the 24-hour movement guidelines could also be seen as practically important for lowering the LBP burden for the society, especially when considering the high prevalence of LBP in the general adult population. However, given the cross-sectional design of our study, these possible practical implications should be taken with caution and confirmed in future studies using a longitudinal or experimental study design.

### 4.4. Strengths and limitations

The key strength of our study is a relatively large sample size that allowed us to explore the associations of meeting all possible combinations of MVPA, SB and sleep recommendations from the 24-hour movement behaviours guidelines with LBP. Another strength of the current study is that we addressed co-dependence between physical activity, SB and sleep, unlike most previous studies that analysed each of the movement behaviours in isolation to the others.

There are also some limitations that need to be mentioned. First, the generalizability of the findings may be somewhat limited due to the way participants were recruited. However, in most sociodemographic characteristics our sample was similar to the general adult population in Slovenia [29]. Second, we relied on self-reported data that could be affected by recall and social desirability biases. Third, we did not conduct a stratified analysis by the severity of LBP [77]–[80]. The associations between movement behaviours and LBP might be different across such strata. However, such an LBP classification typically requires a clinical examination of participants, which was beyond the scope of our study. Fourth, we did not collect data on continuity of experiencing pain (i.e., persistent pain). The associations between 24-hour movement behaviours with persistent pain should be explored in future studies. Finally, our study had a cross-sectional design, which did not allow us to draw conclusions about the causality of the relationships. For example, it might be that higher frequency and intensity of LBP reduce the likelihood of meeting the 24-hour movement guidelines, or the relationship might be bi-directional.

## 5. Conclusions

Meeting the SB and sleep recommendations in combination is associated with a lower likelihood of LBP, while adhering to the overall 24-hour movement guidelines or any combination of recommendations that includes sleep is associated with lower frequency and intensity of LBP among LBP sufferers. These findings may inform future guidelines for the prevention and management of LBP. However, given the possible bi-directional relationships between movement behaviours and LBP, the findings should be considered with caution.

Future studies should evaluate the effectiveness of physical activity, SB and sleep interventions for the prevention and management of LBP. Future studies should also consider assessing movement behaviours using activity monitors, including a wider range of LBP outcomes, conducting stratified analyses by LBP severity and using a longitudinal study design. It may also be worth exploring the optimal quantitative thresholds for movement behaviours to improve LBP outcomes, as they may differ from the thresholds provided in the 24-hour movement guidelines.

## Use of AI tools declaration

The authors declare they have not used Artificial Intelligence (AI) tools in the creation of this article.
