# Supplementary material for: Association of meeting 24-hour movement guidelines with low back pain among adults
Source: AIMS Public Health. 2023 Nov 24;10(4):964–79. doi: 10.3934/publichealth.2023062 (PMC10764968; doi:10.3934/publichealth.2023062)
Supplement: Supplementary file 1 [file publichealth-10-04-062-s001.pdf]

AIMS Public Health

AIMS Public Health, 10(4): 964–979.
DOI: 10.3934/publichealth.2023062
Received: 27 July 2023
Revised: 13 October 2023
Accepted: 09 November 2023
Published: 24 November 2023

http://www.aimspress.com/journal/aimsph

Research article

Association of meeting 24-hour movement guidelines with low back pain among adults

Kaja Kastelic[1,2], Nejc Šarabon[2,3], Michael D. Burnard[1,2], Dean Lipovac[1,2] and Željko Pedišić[4,*]

[1] Andrej Marušič Institute, University of Primorska, Muzejski trg 2, 6000 Koper, Slovenia
[2] InnoRenew CoE, Livade 6a, 6310 Izola, Slovenia
[3] Faculty of Health Sciences, University of Primorska, Polje 42, 6310 Izola, Slovenia
[4] Institute for Health and Sport, Victoria University, Building P, Footscray Park Campus, Ballarat Road, Footscray VIC 3011, Melbourne, Australia

* **Correspondence:** Email: zeljko.pedisic@vu.edu.au; Tel: +61399195275.

Supplementary

Table S1. Participant LBP characteristics (LBP sufferers, *n* = 1660).

Characteristic	LBP sufferers *n* (%)
Frequency of LBP in the past year	
1–30 days with LBP	1211 (73)
31–90 days with LBP	146 (9)
More than 90 days with LBP	303 (18)
Intensity of LBP in the past year	
Mild LBP	1019 (61)
Moderate LBP	401 (24)
Severe LBP	240 (14)
Intensity of LBP in the past month	
No LBP	283 (17)
Mild LBP	776 (47)
Moderate LBP	290 (17)
Severe LBP	311 (19)
Intensity of LBP in the past week	
No LBP	588 (35)
Mild LBP	714 (43)
Moderate LBP	175 (11)
Severe LBP	183 (11)

Note: Abbreviations: LBP = low back pain. Note that intensity of LBP in the past year refer to average LBP intensity, and intensity of LBP in the past month and past week refer to worst LBP intensity.

Table S2: Associations between meeting different combinations of 24-hour movement guidelines and experiencing low back pain in the past year (*n* = 2333).

Variable	Unadjusted model					Adjusted model				
	Estimate	SE	*Odds Ratio* [95% *CI*]	*z*	*p*	Estimate	SE	*Odds Ratio* [95% *CI*]	*z*	*p*
Guideline(s) met										
None	[ref]					[ref]				
Only MVPA	-0.158	0.233	0.854 [0.539, 1.345]	-0.678	0.498	-0.105	0.237	0.900 [0.563, 1.429]	-0.444	0.657
Only SB	-0.261	0.243	0.770 [0.477, 1.238]	-1.076	0.282	-0.260	0.247	0.771 [0.474, 1.251]	-1.051	0.293
Only sleep	-0.338	0.230	0.713 [0.452, 1.116]	-1.467	0.142	-0.198	0.235	0.821 [0.515, 1.297]	-0.841	0.401
For MVPA and SB	-0.246	0.221	0.782 [0.504, 1.201]	-1.112	0.266	-0.148	0.226	0.862 [0.551, 1.337]	-0.656	0.512
For MVPA and sleep	-0.425	0.209	0.654 [0.431, 0.979]	-2.032	0.042	-0.225	0.214	0.799 [0.522, 1.209]	-1.050	0.294
For SB and sleep	-0.169	0.218	0.844 [0.547, 1.289]	-0.775	0.438	-0.076	0.222	0.927 [0.596, 1.427]	-0.342	0.732
For MVPA, SB, and sleep	-0.123	0.200	0.885 [0.592, 1.301]	-0.612	0.541	0.034	0.206	1.035 [0.686, 1.540]	0.168	0.867
Age										
middle						0.329	0.103	1.389 [1.136, 1.700]	3.195	0.001
older						0.256	0.160	1.291 [0.947, 1.773]	1.600	0.110
Sex: male						0.063	0.113	1.065 [0.855, 1.330]	0.559	0.576
BMI: overweight/obese						0.255	0.099	1.291 [1.064, 1.567]	2.590	0.010
Stress: stressed						0.416	0.104	1.515 [1.237, 1.861]	3.991	<0.001
Smoking: no						-0.102	0.126	0.903 [0.702, 1.153]	-0.810	0.418
Education: higer						-0.268	0.112	0.765 [0.613, 0.950]	-2.401	0.016
Socioeconomic status:										
middle						-0.329	0.177	0.720 [0.504, 1.011]	-1.855	0.064
higher						-0.722	0.216	0.486 [0.316, 0.738]	-3.351	0.001
Intercept	1.122	0.178	3.071 [2.189, 4.400]	6.316	<0.001	1.176	0.279	3.241 [1.888, 5.655]	4.207	<0.001

Continued on next page

Variable	Unadjusted model					Adjusted model				
	Estimate	SE	*Odds Ratio* [95% *CI*]	*z*	*p*	Estimate	SE	*Odds Ratio* [95% *CI*]	*z*	*p*
Number of guidelines met										
0	[ref]					[ref]				
1	-0.252	0.198	0.777 [0.522, 1.139]	-1.271	0.204	-0.183	0.202	0.833 [0.556, 1.230]	-0.903	0.366
2	-0.293	0.191	0.746 [0.508, 1.077]	-1.532	0.126	-0.155	0.195	0.856 [0.579, 1.246]	-0.795	0.426
3	-0.123	0.200	0.885 [0.592, 1.301]	-0.612	0.541	0.035	0.206	1.036 [0.687, 1.541]	0.171	0.864
Age:										
middle						0.338	0.102	1.402 [1.149, 1.712]	3.324	0.001
older						0.259	0.158	1.296 [0.953, 1.774]	1.638	0.101
Sex: male						0.057	0.112	1.059 [0.851, 1.321]	0.512	0.608
BMI: overweight/obese						0.261	0.098	1.298 [1.071, 1.575]	2.658	0.008
Stress: stressed						0.417	0.104	1.517 [1.239, 1.863]	4.012	<0.001
Smoking: no						-0.104	0.126	0.901 [0.701, 1.151]	-0.825	0.409
Education: higer						-0.267	0.112	0.766 [0.614, 0.951]	-2.392	0.017
Socioeconomic status:										
middle						-0.329	0.177	0.719 [0.504, 1.010]	-1.859	0.063
higher						-0.721	0.215	0.486 [0.317, 0.738]	-3.349	0.001
Intercept	1.122	0.178	3.071 [2.189, 4.400]	6.316	<0.001	1.170	0.279	3.221 [1.878, 5.618]	4.190	<0.001

Note: Abbreviations: SE, standard error; CI, confidence interval; MVPA = moderate-vigorous physical activity; SB = sedentary behaviour; BMI = body mass index.

Table S3. Associations between meeting different combinations of 24-hour movement guidelines and experiencing low back pain in the past month (*n* = 2333).

Variable	Unadjusted model					Adjusted model				
	Estimate	SE	*Odds Ratio* [95% *CI*]	*z*	*p*	Estimate	SE	*Odds Ratio* [95% *CI*]	*z*	*p*
Guideline(s) met										
None	[ref]					[ref]				
Only MVPA	-0.242	0.213	0.785 [0.516, 1.190]	-1.137	0.255	-0.19	0.217	0.827 [0.539, 1.265]	-0.874	0.382
Only SB	-0.359	0.223	0.699 [0.450, 1.081]	-1.607	0.108	-0.348	0.228	0.706 [0.451, 1.102]	-1.529	0.126
Only sleep	-0.349	0.213	0.706 [0.464, 1.069]	-1.639	0.101	-0.223	0.218	0.800 [0.520, 1.224]	-1.025	0.305
For MVPA and SB	-0.323	0.203	0.724 [0.485, 1.074]	-1.594	0.111	-0.2	0.208	0.818 [0.543, 1.227]	-0.965	0.335
For MVPA and sleep	-0.366	0.193	0.694 [0.473, 1.010]	-1.892	0.059	-0.169	0.198	0.844 [0.570, 1.243]	-0.852	0.394
For SB and sleep	-0.544	0.198	0.580 [0.392, 0.854]	-2.743	0.006	-0.447	0.203	0.640 [0.428, 0.949]	-2.205	0.027
For MVPA, SB, and sleep	-0.299	0.183	0.742 [0.516, 1.057]	-1.637	0.102	-0.118	0.188	0.889 [0.612, 1.281]	-0.626	0.532
Age:										
middle						0.193	0.096	1.213 [1.006, 1.462]	2.018	0.044
older						0.262	0.148	1.299 [0.972, 1.740]	1.762	0.078
Sex: male						-0.019	0.103	0.981 [0.802, 1.201]	-0.188	0.851
BMI: overweight/obese						0.221	0.091	1.248 [1.045, 1.491]	2.441	0.015
Stress: stressed						0.563	0.096	1.756 [1.457, 2.119]	5.889	<0.001
Smoking: no						-0.164	0.116	0.849 [0.675, 1.064]	-1.411	0.158
Education: higer						-0.256	0.101	0.774 [0.635, 0.943]	-2.539	0.011
Socioeconomic status:										
middle						-0.304	0.156	0.738 [0.541, 0.997]	-1.957	0.05
higher						-0.588	0.196	0.556 [0.377, 0.815]	-2.994	0.003
Intercept	0.693	0.162	2 [1.462, 2.766]	4.273	<0.001	0.781	0.253	2.183 [1.335, 3.599]	3.088	0.002

Continued on next page

Variable	Unadjusted model					Adjusted model				
	Estimate	SE	*Odds Ratio* [95% *CI*]	*z*	*p*	Estimate	SE	*Odds Ratio* [95% *CI*]	*z*	*p*
Number of guidelines met										
0	[ref]					[ref]				
1	-0.313	0.182	0.731 [0.509, 1.04]	-1.723	0.085	-0.247	0.186	0.781 [0.540, 1.119]	-1.333	0.183
2	-0.412	0.175	0.663 [0.468, 0.930]	-2.354	0.019	-0.269	0.179	0.764 [0.535, 1.081]	-1.506	0.132
3	-0.299	0.183	0.742 [0.516, 1.057]	-1.637	0.102	-0.118	0.188	0.889 [0.612, 1.280]	-0.628	0.530
Age:										
middle						0.187	0.094	1.206 [1.002, 1.451]	1.982	0.048
older						0.249	0.147	1.283 [0.962, 1.714]	1.693	0.090
Sex: male						-0.005	0.103	0.995 [0.814, 1.218]	-0.045	0.964
BMI: overweight/obese						0.209	0.090	1.232 [1.033, 1.471]	2.314	0.021
Stress: stressed						0.561	0.095	1.753 [1.455, 2.115]	5.886	<0.001
Smoking: no						-0.163	0.116	0.850 [0.676, 1.065]	-1.405	0.160
Education: higer						-0.252	0.101	0.778 [0.638, 0.947]	-2.499	0.012
Socioeconomic status:										
middle						-0.304	0.155	0.738 [0.542, 0.997]	-1.957	0.050
higher						-0.573	0.196	0.564 [0.383, 0.826]	-2.925	0.003
Intercept	0.693	0.162	2 [1.462, 2.766]	4.273	<0.001	0.782	0.252	2.185 [1.338, 3.601]	3.097	0.002

Note: Abbreviations: SE, standard error; CI, confidence interval; MVPA = moderate-vigorous physical activity; SB = sedentary behaviour; BMI = body mass index.

Table S4. Associations between meeting different combinations of 24-hour movement guidelines and experiencing low back pain in the past week (*n* = 2333).

Variable	Unadjusted model					Adjusted model				
	Estimate	SE	*Odds Ratio* [95% *CI*]	*z*	*p*	Estimate	SE	*Odds Ratio* [95% *CI*]	*z*	*p*
Guideline(s) met										
None	[ref]					[ref]				
Only MVPA	-0.209	0.204	0.812 [0.543, 1.211]	-1.021	0.307	-0.165	0.209	0.848 [0.562, 1.278]	-0.787	0.431
Only SB	-0.325	0.216	0.722 [0.472, 1.102]	-1.508	0.132	-0.343	0.221	0.710 [0.459, 1.094]	-1.551	0.121
Only sleep	-0.319	0.205	0.727 [0.485, 1.085]	-1.556	0.120	-0.181	0.211	0.834 [0.551, 1.261]	-0.858	0.391
For MVPA and SB	-0.250	0.195	0.779 [0.531, 1.141]	-1.281	0.200	-0.172	0.200	0.842 [0.568, 1.246]	-0.858	0.391
For MVPA and sleep	-0.527	0.186	0.591 [0.409, 0.850]	-2.826	0.005	-0.316	0.192	0.729 [0.500, 1.061]	-1.647	0.100
For SB and sleep	-0.722	0.194	0.486 [0.332, 0.709]	-3.732	<0.001	-0.659	0.198	0.517 [0.350, 0.762]	-3.324	0.001
For MVPA, SB, and sleep	-0.318	0.174	0.728 [0.516, 1.023]	-1.824	0.068	-0.183	0.18	0.832 [0.584, 1.185]	-1.016	0.309
Age:										
middle						0.425	0.095	1.530 [1.270, 1.845]	4.464	<0.001
older						0.497	0.148	1.644 [1.231, 2.197]	3.364	0.001
Sex: male						-0.147	0.103	0.863 [0.705, 1.055]	-1.435	0.151
BMI: overweight/obese						0.202	0.090	1.224 [1.027, 1.460]	2.254	0.024
Stress: stressed						0.434	0.093	1.543 [1.286, 1.853]	4.650	<0.001
Smoking: no						-0.160	0.113	0.852 [0.683, 1.063]	-1.416	0.157
Education: higer						-0.278	0.098	0.757 [0.625, 0.919]	-2.824	0.005
Socioeconomic status:										
middle						-0.314	0.147	0.730 [0.547, 0.973]	-2.138	0.033
higher						-0.674	0.193	0.510 [0.349, 0.742]	-3.499	<0.001
Intercept	0.199	0.154	1.221 [0.904, 1.653]	1.298	0.194	0.263	0.242	1.301 [0.810, 2.094]	1.087	0.277

Continued on next page

Variable	Unadjusted model					Adjusted model				
	Estimate	SE	*Odds Ratio* [95% *CI*]	*z*	*p*	Estimate	SE	*Odds Ratio* [95% *CI*]	*z*	*p*
Number of guidelines met										
0	[ref]					[ref]				
1	-0.281	0.174	0.755 [0.536, 1.060]	-1.619	0.106	-0.222	0.178	0.801 [0.564, 1.134]	-1.248	0.212
2	-0.507	0.167	0.602 [0.434, 0.835]	-3.033	0.002	-0.384	0.171	0.681 [0.486, 0.953]	-2.240	0.025
3	-0.318	0.174	0.728 [0.516, 1.023]	-1.824	0.068	-0.185	0.180	0.831 [0.583, 1.182]	-1.028	0.304
Age:										
middle						0.427	0.094	1.532 [1.275, 1.843]	4.536	<0.001
older						0.495	0.146	1.641 [1.232, 2.187]	3.385	0.001
Sex: male						-0.129	0.102	0.879 [0.719, 1.073]	-1.266	0.206
BMI: overweight/obese						0.187	0.089	1.205 [1.012, 1.436]	2.094	0.036
Stress: stressed						0.429	0.093	1.536 [1.280, 1.843]	4.616	<0.001
Smoking: no						-0.161	0.113	0.851 [0.682, 1.061]	-1.433	0.152
Education: higer						-0.274	0.098	0.760 [0.627, 0.921]	-2.798	0.005
Socioeconomic status:										
middle						-0.313	0.147	0.731 [0.548, 0.974]	-2.136	0.033
higher						-0.658	0.192	0.518 [0.355, 0.754]	-3.422	0.001
Intercept	0.199	0.154	1.221 [0.904, 1.653]	1.298	0.194	0.265	0.242	1.303 [0.812, 2.095]	1.096	0.273

Note: Abbreviations: SE, standard error; CI, confidence interval; MVPA = moderate-vigorous physical activity; SB = sedentary behaviour; BMI = body mass index.

Table S5. Associations of meeting different combinations of 24-hour movement guidelines with the frequency of low back pain (*n* = 1660).

Variable	Unadjusted model					Adjusted model				
	Estimate	SE	*Odds Ratio* [95% *CI*]	*t*	*p*	Estimate	SE	*Odds Ratio* [95% *CI*]	*t*	*p*
Guideline(s) met										
None	[ref]					[ref]				
Only MVPA	-0.249	0.247	0.779 [0.480, 1.266]	-1.010	0.313	-0.226	0.252	0.798 [0.486, 1.311]	-0.893	0.372
Only SB	-0.141	0.263	0.869 [0.517, 1.455]	-0.536	0.592	-0.174	0.269	0.840 [0.495, 1.423]	-0.648	0.517
Only sleep	-0.366	0.253	0.694 [0.421, 1.140]	-1.443	0.149	-0.232	0.260	0.793 [0.476, 1.322]	-0.891	0.373
For MVPA and SB	-0.278	0.239	0.757 [0.474, 1.212]	-1.165	0.244	-0.213	0.245	0.808 [0.500, 1.311]	-0.866	0.386
For MVPA and sleep	-0.900	0.244	0.406 [0.252, 0.655]	-3.697	<0.001	-0.724	0.249	0.485 [0.297, 0.790]	-2.908	0.004
For SB and sleep	-0.556	0.239	0.573 [0.359, 0.918]	-2.325	0.020	-0.490	0.245	0.613 [0.379, 0.993]	-1.995	0.046
For MVPA, SB, and sleep	-0.634	0.215	0.530 [0.349, 0.813]	-2.943	0.003	-0.493	0.223	0.611 [0.396, 0.949]	-2.217	0.027
Age:										
middle						0.460	0.130	1.584 [1.229, 2.048]	3.529	<0.001
older						0.998	0.195	2.712 [1.849, 3.970]	5.126	<0.001
Sex: male						-0.332	0.140	0.718 [0.543, 0.942]	-2.366	0.018
BMI: overweight/obese						0.072	0.117	1.075 [0.854, 1.353]	0.612	0.540
Stress: stressed						0.644	0.120	1.903 [1.504, 2.411]	5.355	<0.001
Smoking: no						-0.138	0.143	0.871 [0.660, 1.158]	-0.962	0.336
Education: higer						-0.089	0.126	0.915 [0.715, 1.173]	-0.704	0.482
Socioeconomic status:										
middle						-0.318	0.172	0.728 [0.521, 1.024]	-1.847	0.065
higher						-0.544	0.249	0.581 [0.354, 0.944]	-2.179	0.029
Intercepts:										
1–30 days|31–90 days	0.532	0.184		2.892	0.004	0.743	0.298		2.497	0.013
31–90 days|more than 90 days	1.046	0.186		5.623	<0.001	1.275	0.299		4.267	<0.001

Continued on next page

Variable	Unadjusted model					Adjusted model				
	Estimate	SE	*Odds Ratio* [95% *CI*]	*t*	*p*	Estimate	SE	*Odds Ratio* [95% *CI*]	*t*	*p*
Number of guidelines met										
0	[ref]					[ref]				
1	-0.259	0.210	0.772 [0.514, 1.171]	-1.234	0.217	-0.213	0.215	0.808 [0.533, 1.237]	-0.994	0.320
2	-0.586	0.204	0.557 [0.375, 0.835]	-2.869	0.004	-0.482	0.210	0.618 [0.411, 0.937]	-2.298	0.022
3	-0.634	0.215	0.531 [0.349, 0.813]	-2.942	0.003	-0.499	0.223	0.607 [0.394, 0.944]	-2.241	0.025
Age:										
middle						0.488	0.129	1.629 [1.268, 2.101]	3.79	<0.001
older						1.045	0.192	2.843 [1.947, 4.143]	5.428	<0.001
Sex: male						-0.342	0.139	0.710 [0.539, 0.930]	-2.457	0.014
BMI: overweight/obese						0.078	0.117	1.081 [0.860, 1.361]	0.668	0.504
Stress: stressed						0.635	0.119	1.888 [1.494, 2.387]	5.319	<0.001
Smoking: no						-0.143	0.143	0.867 [0.657, 1.151]	-0.998	0.318
Education: higer						-0.096	0.126	0.908 [0.711, 1.164]	-0.764	0.445
Socioeconomic status:										
middle						-0.309	0.172	0.734 [0.526, 1.032]	-1.802	0.072
higher						-0.548	0.249	0.578 [0.353, 0.938]	-2.205	0.027
Intercepts:										
1–30 days|31–90 days	0.533	0.184	NA [NA, NA]	2.896	0.004	0.757	0.297		2.554	0.011
31–90 days|more than 90 days	1.044	0.186	NA [NA, NA]	5.615	<0.001	1.288	0.298		4.326	<0.001

Note: Abbreviations: SE, standard error; CI, confidence interval; MVPA = moderate-vigorous physical activity; SB = sedentary behaviour; BMI = body mass index. The analyses included only LBP sufferers.

Table S6. Associations of meeting different combinations of 24-hour movement guidelines with average low back pain intensity in the past year (*n* = 1660).

Variable	Unadjusted model					Adjusted model				
	Estimate	SE	*Odds Ratio* [95% *CI*]	*t*	*p*	Estimate	SE	*Odds Ratio* [95% *CI*]	*t*	*p*
Guideline(s) met										
None	[ref]					[ref]				
Only MVPA	-0.100	0.226	0.905 [0.581, 1.409]	-0.444	0.657	-0.103	0.230	0.902 [0.574, 1.417]	-0.449	0.653
Only SB	-0.161	0.240	0.851 [0.531, 1.363]	-0.670	0.503	-0.225	0.244	0.799 [0.494, 1.289]	-0.919	0.358
Only sleep	-0.444	0.236	0.641 [0.403, 1.018]	-1.882	0.060	-0.418	0.242	0.659 [0.409, 1.058]	-1.723	0.085
For MVPA and SB	-0.315	0.217	0.730 [0.477, 1.118]	-1.451	0.147	-0.257	0.222	0.773 [0.501, 1.195]	-1.160	0.246
For MVPA and sleep	-0.455	0.210	0.635 [0.421, 0.958]	-2.171	0.030	-0.320	0.214	0.726 [0.478, 1.107]	-1.494	0.135
For SB and sleep	-0.658	0.219	0.518 [0.337, 0.795]	-3.009	0.003	-0.646	0.224	0.524 [0.338, 0.813]	-2.884	0.004
For MVPA, SB, and sleep	-0.534	0.193	0.586 [0.402, 0.858]	-2.763	0.006	-0.440	0.199	0.644 [0.437, 0.954]	-2.207	0.027
Age:										
middle						0.068	0.114	1.071 [0.857, 1.339]	0.601	0.548
older						0.568	0.173	1.764 [1.256, 2.472]	3.289	0.001
Sex: male						-0.414	0.123	0.661 [0.518, 0.839]	-3.369	0.001
BMI: overweight/obese						0.414	0.106	1.512 [1.230, 1.861]	3.917	<0.001
Stress: stressed						0.446	0.108	1.562 [1.264, 1.930]	4.134	<0.001
Smoking: no						0.152	0.131	1.164 [0.902, 1.510]	1.159	0.247
Education: higer						-0.271	0.112	0.763 [0.613, 0.950]	-2.426	0.015
Socioeconomic status:										
middle						-0.448	0.156	0.639 [0.471, 0.869]	-2.869	0.004
higher						-0.785	0.228	0.456 [0.291, 0.712]	-3.438	0.001
Intercepts:										
mild LBP|moderate LBP	0.075	0.167		0.449	0.653	0.025	0.266		0.095	0.925
moderate LBP|severe LBP	1.399	0.172		8.132	<0.001	1.399	0.269		5.208	<0.001

Continued on next page

Variable	Unadjusted model					Adjusted model				
	Estimate	SE	*Odds Ratio* [95% *CI*]	*t*	*p*	Estimate	SE	*Odds Ratio* [95% *CI*]	*t*	*p*
Number of guidelines met										
0	[ref]					[ref]				
1	-0.231	0.191	0.794 [0.547, 1.157]	-1.209	0.226	-0.238	0.195	0.788 [0.539, 1.158]	-1.220	0.223
2	-0.476	0.184	0.621 [0.434, 0.894]	-2.588	0.010	-0.399	0.188	0.671 [0.465, 0.972]	-2.125	0.034
3	-0.534	0.193	0.586 [0.402, 0.858]	-2.761	0.006	-0.434	0.199	0.648 [0.439, 0.960]	-2.178	0.029
Age:										
middle						0.088	0.113	1.092 [0.876, 1.362]	0.782	0.434
older						0.572	0.171	1.771 [1.266, 2.472]	3.350	0.001
Sex: male						-0.388	0.122	0.679 [0.533, 0.861]	-3.172	0.002
BMI: overweight/obese						0.404	0.105	1.498 [1.219, 1.842]	3.842	<0.001
Stress: stressed						0.458	0.107	1.581 [1.281, 1.951]	4.265	<0.001
Smoking: no						0.148	0.131	1.159 [0.898, 1.503]	1.125	0.260
Education: higer						-0.269	0.111	0.764 [0.614, 0.951]	-2.414	0.016
Socioeconomic status:										
middle						-0.444	0.156	0.641 [0.473, 0.872]	-2.848	0.004
higher						-0.777	0.228	0.460 [0.293, 0.717]	-3.411	0.001
Intercepts:										
mild LBP|moderate LBP	0.076	0.167		0.454	0.649	0.049	0.265		0.184	0.854
moderate LBP|severe LBP	1.397	0.172		8.121	<0.001	1.419	0.268		5.296	<0.001

Note: Abbreviations: SE, standard error; CI, confidence interval; MVPA = moderate-vigorous physical activity; SB = sedentary behaviour; BMI = body mass index; LBP = low back pain. The analyses included only LBP sufferers.

Table S7. Associations of meeting different combinations of 24-hour movement guidelines with highest low back pain intensity in the past month (*n* = 1660).

Variable	Unadjusted model					Adjusted model				
	Estimate	SE	*Odds Ratio* [95% *CI*]	*t*	*p*	Estimate	SE	*Odds Ratio* [95% *CI*]	*t*	*p*
Guideline(s) met										
None	[ref]					[ref]				
Only MVPA	-0.159	0.219	0.853 [0.555, 1.311]	-0.725	0.469	-0.152	0.221	0.859 [0.557, 1.324]	-0.690	0.490
Only SB	-0.126	0.232	0.881 [0.559, 1.389]	-0.544	0.587	-0.145	0.235	0.865 [0.546, 1.370]	-0.618	0.537
Only sleep	-0.182	0.221	0.834 [0.540, 1.287]	-0.821	0.411	-0.153	0.224	0.859 [0.553, 1.332]	-0.681	0.496
For MVPA and SB	-0.158	0.210	0.854 [0.566, 1.290]	-0.750	0.453	-0.074	0.213	0.928 [0.612, 1.408]	-0.350	0.727
For MVPA and sleep	-0.229	0.199	0.795 [0.538, 1.176]	-1.147	0.251	-0.124	0.202	0.884 [0.595, 1.313]	-0.612	0.540
For SB and sleep	-0.742	0.206	0.476 [0.317, 0.713]	-3.596	<0.001	-0.690	0.208	0.502 [0.333, 0.755]	-3.310	0.001
For MVPA, SB, and sleep	-0.500	0.186	0.607 [0.421, 0.873]	-2.693	0.007	-0.380	0.189	0.684 [0.472, 0.990]	-2.015	0.044
Age:										
middle						-0.033	0.103	0.967 [0.791, 1.183]	-0.325	0.746
older						0.398	0.163	1.489 [1.083, 2.048]	2.451	0.014
Sex: male						-0.199	0.109	0.820 [0.661, 1.015]	-1.819	0.069
BMI: overweight/obese						0.160	0.096	1.173 [0.972, 1.415]	1.666	0.096
Stress: stressed						0.555	0.099	1.741 [1.434, 2.116]	5.593	<0.001
Smoking: no						-0.043	0.118	0.958 [0.761, 1.208]	-0.361	0.718
Education: higer						-0.255	0.104	0.775 [0.632, 0.950]	-2.454	0.014
Socioeconomic status:										
middle						-0.411	0.151	0.663 [0.493, 0.891]	-2.727	0.006
higher						-0.424	0.205	0.654 [0.438, 0.977]	-2.071	0.038
Intercepts:										
no LBP|mild LBP	-1.922	0.171		-11.222	<0.001	-2.207	0.259		-8.52	<0.001
mild LBP|moderate LBP	0.250	0.164		1.522	0.128	0.024	0.253		0.094	0.925
moderate LBP|severe LBP	1.158	0.167		6.925	<0.001	0.959	0.255		3.759	<0.001

Continued on next page

Variable	Unadjusted model					Adjusted model				
	Estimate	SE	*Odds Ratio* [95% *CI*]	*t*	*p*	Estimate	SE	*Odds Ratio* [95% *CI*]	*t*	*p*
Number of guidelines met										
0	[ref]					[ref]				
1	-0.157	0.185	0.855 [0.595, 1.229]	-0.849	0.396	-0.147	0.186	0.864 [0.599, 1.245]	-0.787	0.431
2	-0.374	0.178	0.688 [0.485, 0.975]	-2.106	0.035	-0.292	0.180	0.747 [0.525, 1.062]	-1.628	0.104
3	-0.498	0.185	0.608 [0.423, 0.874]	-2.685	0.007	-0.375	0.188	0.688 [0.475, 0.995]	-1.988	0.047
Age:										
middle						-0.04	0.101	0.961 [0.788, 1.172]	-0.394	0.693
older						0.374	0.160	1.454 [1.062, 1.989]	2.337	0.019
Sex: male						-0.167	0.109	0.846 [0.684, 1.047]	-1.534	0.125
BMI: overweight/obese						0.142	0.095	1.153 [0.956, 1.390]	1.493	0.135
Stress: stressed						0.557	0.099	1.746 [1.439, 2.120]	5.644	< 0.001
Smoking: no						-0.043	0.118	0.958 [0.761, 1.207]	-0.362	0.718
Education: higer						-0.254	0.104	0.775 [0.633, 0.950]	-2.454	0.014
Socioeconomic status:										
middle						-0.406	0.151	0.666 [0.496, 0.895]	-2.699	0.007
higher						-0.418	0.204	0.658 [0.441, 0.982]	-2.048	0.041
Intercepts:										
no LBP|mild LBP	-1.912	0.171		-11.184	<0.001	-2.193	0.258		-8.503	<0.001
mild LBP|moderate LBP	0.248	0.164		1.512	0.131	0.023	0.252		0.092	0.927
moderate LBP|severe LBP	1.153	0.167		6.903	<0.001	0.954	0.254		3.757	<0.001

Note: Abbreviations: SE, standard error; CI, confidence interval; MVPA = moderate-vigorous physical activity; SB = sedentary behaviour; BMI = body mass index; LBP = low back pain. The analyses included only LBP sufferers.

Table S8. Associations of meeting different combinations of 24-hour movement guidelines with highest low back pain intensity in the past week (*n* = 1660).

Variable	Unadjusted model					Adjusted model				
	Estimate	SE	*Odds Ratio* [95% *CI*]	*t*	*p*	Estimate	SE	*Odds Ratio* [95% *CI*]	*t*	*p*
Guideline(s) met										
None	[ref]					[ref]				
Only MVPA	-0.216	0.220	0.806 [0.523, 1.241]	-0.980	0.327	-0.207	0.222	0.813 [0.525, 1.257]	-0.933	0.351
Only SB	-0.185	0.237	0.831 [0.522, 1.321]	-0.783	0.434	-0.256	0.239	0.774 [0.485, 1.236]	-1.072	0.284
Only sleep	-0.237	0.223	0.789 [0.509, 1.222]	-1.062	0.288	-0.180	0.226	0.835 [0.536, 1.302]	-0.795	0.427
For MVPA and SB	-0.245	0.210	0.783 [0.519, 1.181]	-1.169	0.242	-0.241	0.212	0.786 [0.519, 1.192]	-1.133	0.257
For MVPA and sleep	-0.556	0.202	0.573 [0.386, 0.852]	-2.756	0.006	-0.423	0.205	0.655 [0.438, 0.980]	-2.061	0.039
For SB and sleep	-0.930	0.209	0.395 [0.262, 0.594]	-4.459	<0.001	-0.935	0.212	0.393 [0.259, 0.594]	-4.416	<0.001
For MVPA, SB, and sleep	-0.550	0.186	0.577 [0.400, 0.831]	-2.957	0.003	-0.487	0.190	0.614 [0.423, 0.892]	-2.562	0.01
Age:										
middle						0.322	0.104	1.379 [1.125, 1.692]	3.092	0.002
older						0.743	0.165	2.102 [1.521, 2.906]	4.497	<0.001
Sex: male						-0.368	0.110	0.692 [0.557, 0.859]	-3.332	0.001
BMI: overweight/obese						0.165	0.097	1.180 [0.976, 1.426]	1.709	0.088
Stress: stressed						0.428	0.101	1.534 [1.259, 1.868]	4.252	<0.001
Smoking: no						-0.098	0.120	0.907 [0.717, 1.147]	-0.819	0.413
Education: higer						-0.269	0.105	0.764 [0.622, 0.939]	-2.563	0.010
Socioeconomic status:										
middle						-0.308	0.150	0.735 [0.547, 0.987]	-2.050	0.040
higher						-0.600	0.207	0.549 [0.366, 0.823]	-2.897	0.004
Intercepts:										
no LBP|mild LBP	-1.047	0.167		-6.274	<0.001	-1.203	0.256		-4.699	<0.001
mild LBP|moderate LBP	0.872	0.166		5.243	<0.001	0.781	0.255		3.061	0.002
moderate LBP|severe LBP	1.677	0.173		9.697	<0.001	1.609	0.259		6.211	<0.001

Continued on next page

Variable	Unadjusted model					Adjusted model				
	Estimate	SE	*Odds Ratio* [95% *CI*]	*t*	*p*	Estimate	SE	*Odds Ratio* [95% *CI*]	*t*	*p*
Number of guidelines met										
0	[ref]					[ref]				
1	-0.214	0.186	0.807 [0.560, 1.164]	-1.148	0.251	-0.208	0.188	0.812 [0.562, 1.175]	-1.106	0.269
2	-0.581	0.179	0.559 [0.394, 0.794]	-3.249	0.001	-0.530	0.182	0.589 [0.412, 0.841]	-2.919	0.004
3	-0.548	0.186	0.578 [0.402, 0.832]	-2.948	0.003	-0.483	0.190	0.617 [0.425, 0.895]	-2.544	0.011
Age:										
middle						0.325	0.103	1.385 [1.132, 1.694]	3.168	0.002
older						0.730	0.163	2.075 [1.509, 2.855]	4.487	<0.001
Sex: male						-0.339	0.110	0.713 [0.575, 0.883]	-3.091	0.002
BMI: overweight/obese						0.156	0.096	1.168 [0.968, 1.411]	1.620	0.105
Stress: stressed						0.423	0.100	1.526 [1.255, 1.857]	4.225	<0.001
Smoking: no						-0.103	0.119	0.902 [0.714, 1.140]	-0.862	0.389
Education: higer						-0.264	0.105	0.768 [0.626, 0.943]	-2.520	0.012
Socioeconomic status:										
middle						-0.313	0.150	0.731 [0.545, 0.982]	-2.082	0.037
higher						-0.599	0.207	0.549 [0.366, 0.823]	-2.897	0.004
Intercepts:										
no LBP|mild LBP	-1.041	0.167		-6.246	<0.001	-1.197	0.255		-4.691	<0.001
mild LBP|moderate LBP	0.867	0.166		5.221	<0.001	0.774	0.254		3.046	0.002
moderate LBP|severe LBP	1.670	0.173		9.672	<0.001	1.601	0.258		6.200	<0.001

Note: Abbreviations: SE, standard error; CI, confidence interval; MVPA = moderate-vigorous physical activity; SB = sedentary behaviour; BMI = body mass index; LBP = low back pain. The analyses included only LBP sufferers.

AIMS Press

© 2023 the Author(s), licensee AIMS Press. This is an open access article distributed under the terms of the Creative Commons Attribution License (http://creativecommons.org/licenses/by/4.0)
